# In vitro studies of factors affecting debridement of dental implants by tricalcium phosphate powder abrasive treatment

**DOI:** 10.1038/s41598-023-35053-3

**Published:** 2023-05-22

**Authors:** I-Cheng Chen, Chen-Ying Su, Jia-Jun Tu, Daniel Wenkai Kao, Hsu-Wei Fang

**Affiliations:** 1 Accelerator for Happiness and Health Industry, National Taipei University of Technology, No. 1, Sec. 3, Zhongxiao E. Rd., Taipei, 10608 Taiwan; 2 Department of Chemical Engineering and Biotechnology, National Taipei University of Technology, No. 1, Sec. 3, Zhongxiao E. Rd., Taipei, 10608 Taiwan; 3 Washington Dental Group, No. 111, Sec. 3, Heping E. Rd., Da-an Dist., Taipei City, 10675 Taiwan; 4 Department of Dentistry, National Yang Ming University, No. 155, Yixin Rd., Beitou Dist., Taipei City, 11221 Taiwan; 5 Harvard School of Dental Medicine, Boston, 188 Longwood Ave, Boston, MA 02115 USA; 6 Institute of Biomedical Engineering and Nanomedicine, National Health Research Institutes, No. 35, Keyan Road, Zhunan Town, Miaoli County, 35053 Taiwan; 7 High-value Biomaterials Research and Commercialization Center, National Taipei University of Technology, No. 1, Sec. 3, Zhongxiao E. Rd., 10608 Taipei, Taiwan

**Keywords:** Dental diseases, Chemical engineering, Dentistry

## Abstract

Peri-implantitis is a common complication characterized by inflammation in tissues surrounding dental implants due to plaque accumulation, which can lead to implant failure. While air flow abrasive treatment has been found to be effective for debriding implant surfaces, little is known about the factors that affect its cleaning capacity. This study systematically examined the cleaning capacity of air powder abrasive (APA) treatment with β-tricalcium phosphate (β-TCP) powder, using various powder jetting strengths and different particle sizes. Three sizes of β-TCP powder (S, M, and L) were prepared, and different powder settings (low, medium, and high) were tested. The cleaning capacity was determined by quantifying ink removal, which simulated biofilm removal from the implant surfaces at different time points. The results of the systematic comparisons showed that the most efficient cleaning of implant surfaces was achieved using size M particles with medium setting. Additionally, the amount of powder consumed was found to be critical to cleaning efficiency, and the implant surfaces were altered in all tested groups. These systematically analyzed outcomes may provide insights into the development of potential non-surgical strategies for treating peri-implant diseases.

## Introduction

As dental implants are routine parts in dentistry to support dental prosthesis and replace missing teeth for restorative purpose, the occurrence of peri-implant diseases is increasing. Biological complications such as peri-implant infections are the leading cause of dental implantation failures^1–4^. Peri-implantitis is a very common complication characterized by inflammation in tissues with plaque accumulation around dental implants, and its prevalence varied ranging from 4.7 to 45% at the patient level with various diagnostic criteria^2,5–10^. Peri-implantitis usually displays a more acute inflammatory status following peri-implant mucositis and destructively affects soft and hard tissues, resulting in progressive loss of supporting bone. Various factors may increase the risks of developing peri-implantitis including systematic factors (e.g. smoking, diseases), oral-related factors (e.g. cleaning skills, periodontitis, keratinized mucosa, occlusal overload) or implant-related factors (e.g. implant surface, position and design of the implant-abutment junction, surgical procedure, fixation type)^10^.

Treatments of peri-implantitis to remove biofilm are critical to the longevity of implants, and current managements include surgical and non-surgical procedures. Access flap surgery, resective surgery and bone graft substitute surgery with barrier membranes^11^ have been proposed to control the progression of the peri-implant diseases. For non-surgical treatment, a number of studies have shown different tools for debridement of implant surfaces to control the infection. Low-energy laser irradiation to decontaminate implant surfaces and remove biofilm by multiple passages seemed to be a promising approach^12–14^. Several protocols for mechanical debridement with curettes, ultrasonic devices, or air abrasives were also reported^15–17^. Among them, air flow devices were found to be efficient for the debridement of the implant surfaces^16,18–21^.

The air powder abrasive (APA) device is usually introduced with powder jetted through the nozzle by compressed air to remove plaque or biofilm. Sodium bicarbonate, glycine, erythritol, and calcium phosphate powders are commonly used in dentistry because of their great accessibility, safety and bactericidal property, therefore, the efficiency of cleaning dental implant surfaces with various powders have been discussed in several in vitro and in vivo studies^16,22–25^. Using air-flow device with glycine powder allowed for access and cleaning in the larger defects in an in vitro peri-implantitis model^16^. Air polishing with sodium bicarbonate has been reported to be effective in removing viable bacterial cells and plaque on the implant surface^26^. A systematic review was reported in the in vivo models of peri-implant diseases and the meta-analysis indicated that the treatment outcomes by glycine powder polishing were improved at mucositis sites and it increased the efficacy of non-surgical treatment of peri-implantitis^27^. Karaca et al. conducted an in vitro study and revealed that the air abrasive device with trehalose powder presented a better performance than the Er:YAG laser^28^. In addition, APA with hydroxylapatite (HA) and calcium phosphate (tricalcium Phosphate, TCP) showed 99% biofilm removal from titanium discs and caused minimal changes to the surface. Furthermore, cleaning effects of APA were also tested with ex-planted human implants, and the study showed positive outcome that the large calculus was removed by APA treatment with osteoconductive powder (HA and calcium phosphate)^23,24^. Altogether, APA treatment could be promising and safe when used in vivo.

Few studies have shown that residues of glycine particles may be left on the surface after APA treatment, potentially causing inefficient osseointegration and disturbing cell response^29–33^. Instead, osteoconductive β-TCP which is beneficial to osseointegration was selected for this study as powder for APA treatment because it is the biocompatible, biodegradable, and highly porous material which can transform to HA, the dominant mineral phase of vertebral tooth and bone tissue, under in vivo conditions^34–36^.

There are yet no publications that have evaluated the efficiency of implant cleaning by APA treatment through systematic comparisons of different parameters. The main objective of this study is to gain a better understanding of the cleaning effects of β-TCP powder APA treatment by examining various factors in an in vitro model, including the jetting strength of powder settings and the sizes of β-TCP particles. Additionally, the study observed and discussed the surface changes of implants after APA treatment. We believe that this study could provide a step forward in preventing future breakdown and managing peri-implantitis and related diseases.

## Materials and methods

### Preparation of β-tri-calcium phosphate β-TCP powder

**Figure 1:**
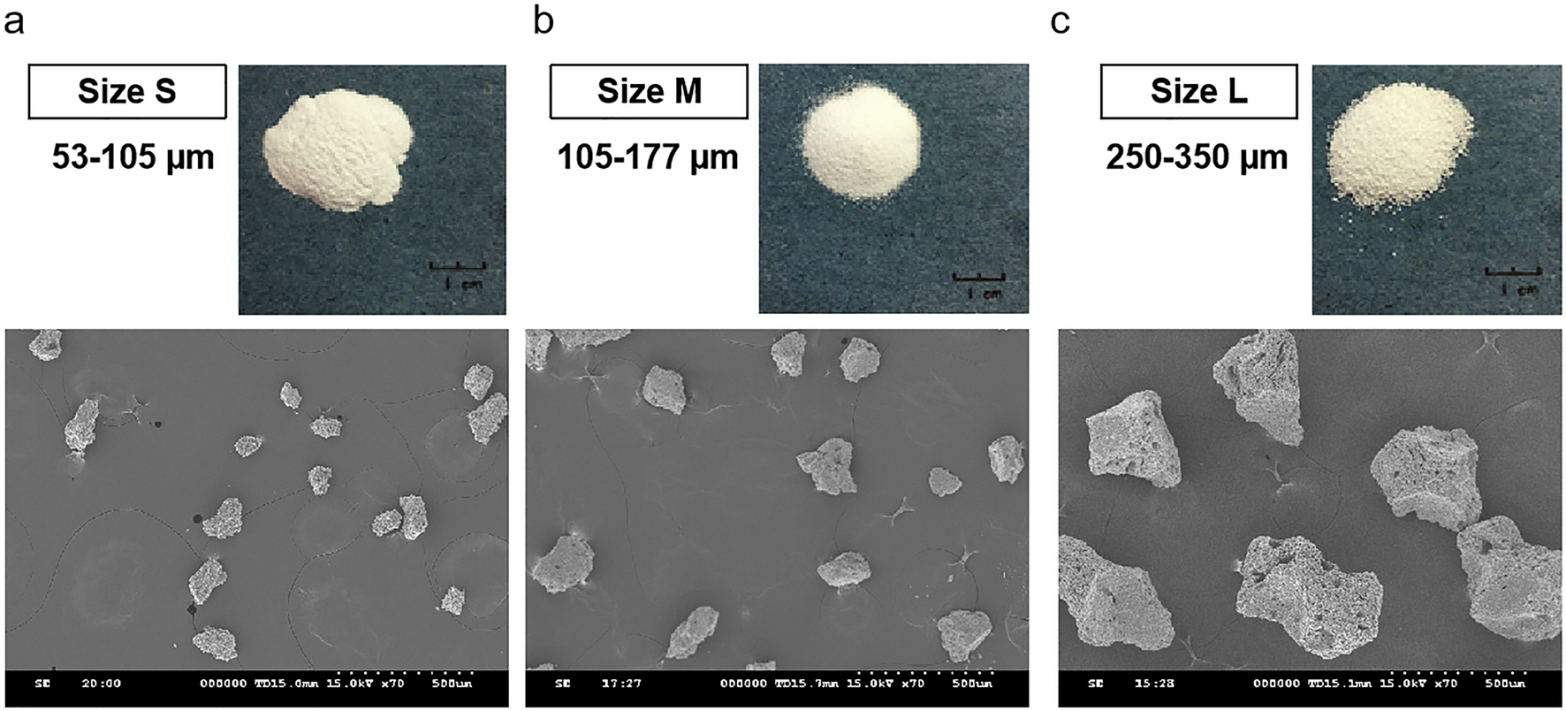
Images of β-TCP powder used in this study with different sizes: (**a**) size S: 53 to 105 μm; (**b**) size M: 105 to 117 μm; (**c**) size L: 250 to 350 μm.

The β-TCP powder was provided by Wiltrom Co., Ltd. (Osteocera Dental Bone Graft, Hsinchu County, Taiwan) and the original particle size was around 1 to 2 mm. β-TCP powder was milled by hand and dried in a 37 °C oven for 12 h. After sieving by meshes with different pore sizes (45, 60, 80, 140, and 270 μm), three groups of particles were obtained for further experiments: (1) size S: particle sizes ranging from 53 to 105 μm; (2) size M: particle sizes ranging from 105 to 117 μm; (3) size L: particle sizes ranging from 250 to 350 μm (Fig. 1).

### Measurement of the cleaning strength

An air powder abrasive device (Prophy-APII, Apoza Enterprise Co., Ltd, New Taipei city, Taiwan) was used with β-TCP powder with fixed water flow at low, medium or high settings for jetting. Size S, M, L of β-TCP powders or glycine (25 μm, kindly provided by Dr. Daniel Wenkai Kao) were filled in the powder tank and jetted from the nozzle with a distance of 3 mm to the aluminum surface for 20 s. The jetting strength was measured by Ultra Small-capacity Load Cell (LTS-100GA-1N, Kyowa, Tokyo, Japan) and recorded by Datalogger (midi LOGGER GL240, GRAPHTEC, Yokohama, Japan).

### Measurement of consumed β-TCP powder

The airflow nozzle was fixed and jetted with a distance of 3 mm to a beaker for 60 s with various jetting strength and different particle sizes. The jetted powder was collected and dried in a 37 °C oven for 12 h then measured by a balance.

### Preparation of the implants

**Figure 2:**
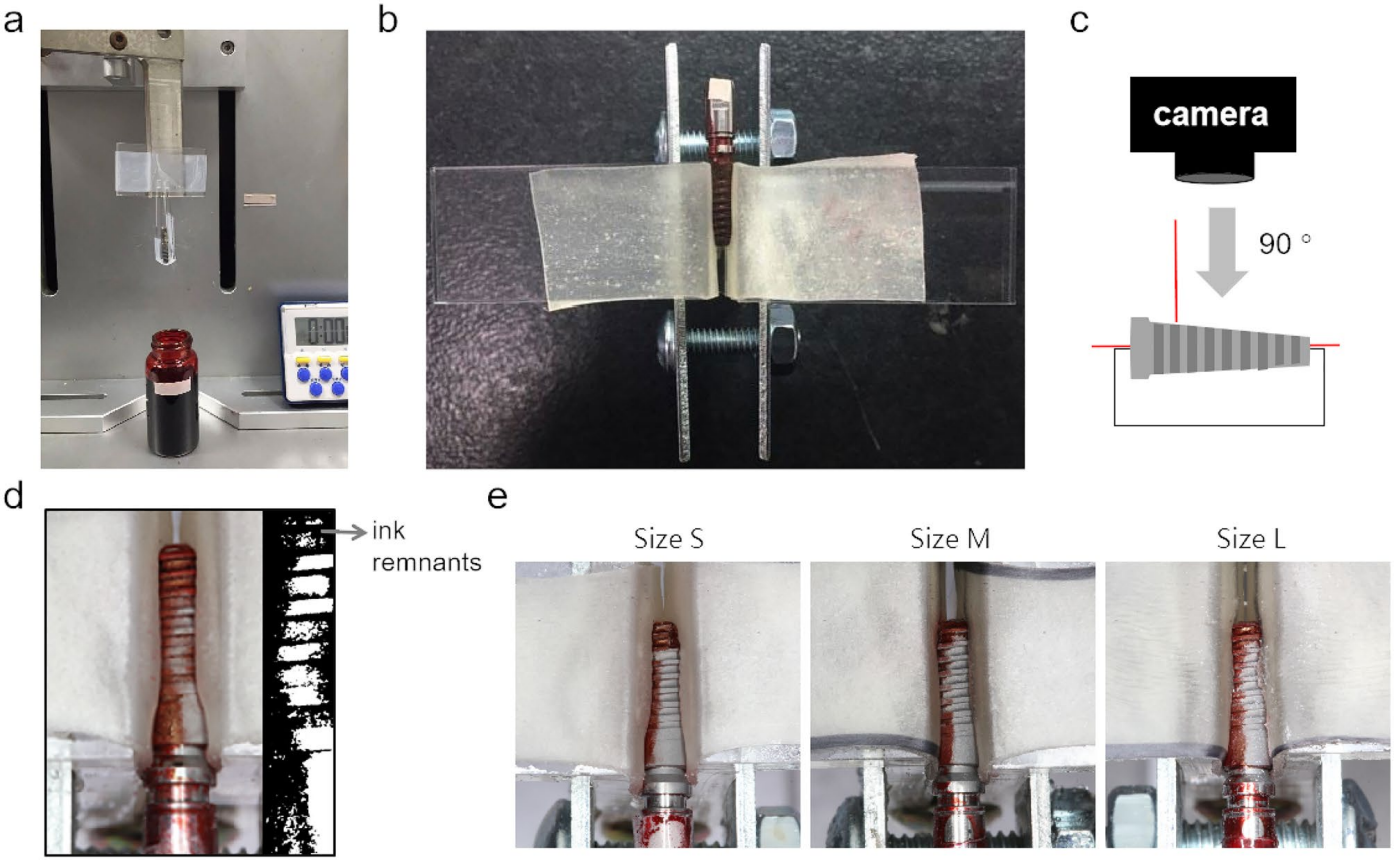
Settings for in vitro model used in this study: (**a**) implant coated with permanent red ink before APA treatment; (**b**) the customized acrylic holder for fixing the stained implant; (**c**) schematic illustration of the image taking vertically to the implant axis; (**d**) digital photograph of treated implant and residual dye determination; (**e**) representative photographs of implants cleaned by different sizes of β-TCP particles for 180 s by APA treatment.

Implants (3.5 mm diameter, 9.5 mm height, Ankylos, DENTSPLY Implants, North Carolina, USA) were dip-coated with permanent red ink (Staedler permanent Lumocolor, Nürnberg, Germany) for 10 s to simulate biofilm coated implant surfaces then air dried for 48 h in a hood (Fig. 2a).

### Air polish abrasive treatment

The dip-coated implants were fixed in a customized acrylic splint and treated with the air powder abrasive device mentioned above with β-TCP powder at different powder settings with fixed water flow (Fig. 2b). The air prophy tip was applied at 3 mm distance with angulation of 90° to the surface for 10, 20, 30, 40, 50, 60, 80, 100, 120 and 180 s.

### Determination of the implant cleaning surface

After the cleaning procedure, the implants were removed from the holder and rinsed with water to remove loosened ink debris. Digital photos were taken by a digital camera (Canon EOS 60D) positioned vertically to the implant axis (Fig. 2c). The dye remnants of the cleaned surface as shown in Fig. 2d were determined and quantified by image processing software Adobe Photoshop CS6 (Adobe systems software, California, USA) and Visual Studio 2015 (Microsoft, Washington, USA). Figure 2e showed the representative photographs of implants treated by different sizes of β-TCP particles for 180 s.

### Scanning electron microscope (SEM)

Implants were examined by SEM using an S-3000H microscope (Hitachi, Tokyo, Japan) under low vacuum conditions. Each specimen was covered with gold by a sputter coater (Ion Sputter E101, Hitachi). Representative images were taken before and after treatment.

### Statistics

For statistical analysis, differences between groups were evaluated by Kruskal–Wallis test and Dunn’s Test, and considered statistically significant at the *p* value less than 0.05.

## Results

### Measurements of cleaning strength and consumed powder amount for APA treatment

**Figure 3:**
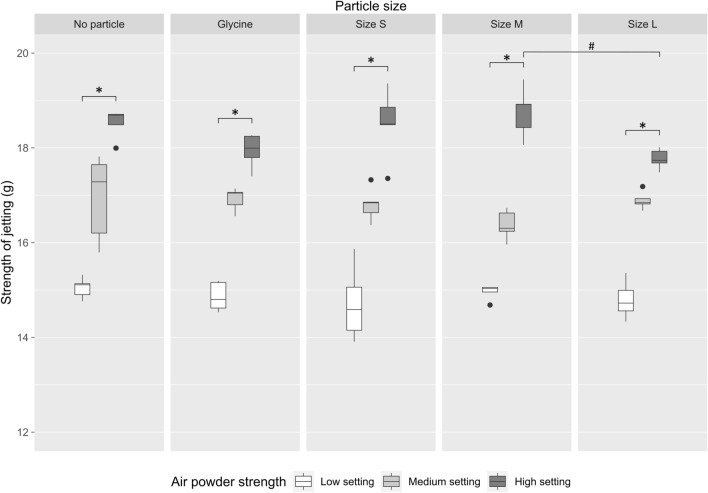
Measurements of jetting strength from low (white boxes), medium (light gray boxes) or high (dark gray boxes) powder settings with different sizes of β-TCP powder and glycine. *p < 0.05 when compared between different settings; ^#^*p* < 0.05 when compared between different particle sizes.

The purpose of this study is to examine various parameters for accessing the optimized cleaning effects to remove biofilm from dental implants by APA treatment. Firstly, the jetting strength of the air abrasive device ("low," "medium," and "high" powder settings) was determined by the load cell to measure small loads in the vertical direction to the surface. When comparing the different strength settings within each size group (no particle, glycine, size S, size M, size L of β-TCP powder), Kruskal–Wallis test showed that for all sizes the strength settings have statistically different data distributions with the same trend (high setting > medium setting > low setting, all *p*-values < 0.05). Dunn’s test showed that only differences between low and high strength settings occurred (*p*-values < 0.05); for other settings, no statistically significant differences were found (*p*-values > 0.05) (Fig. 3).

In terms of air powder strength content, Kruskal–Wallis test results showed that there was no statistically significant differences between different sizes for low and medium strength settings (low settings, white boxes: 14.71 g ~ 15.05 g; medium settings, light gray boxes: 16.37 g ~ 16.95 g; all *p*-values > 0.05; Fig. 3). Interestingly, at high setting group (dark gray boxes, Fig. 3), the jetting strength was notably lower in size L samples than size M group (size L vs. size M: 17.77 g vs. 18.75 g, *p*-values < 0.05).

**Figure 4:**
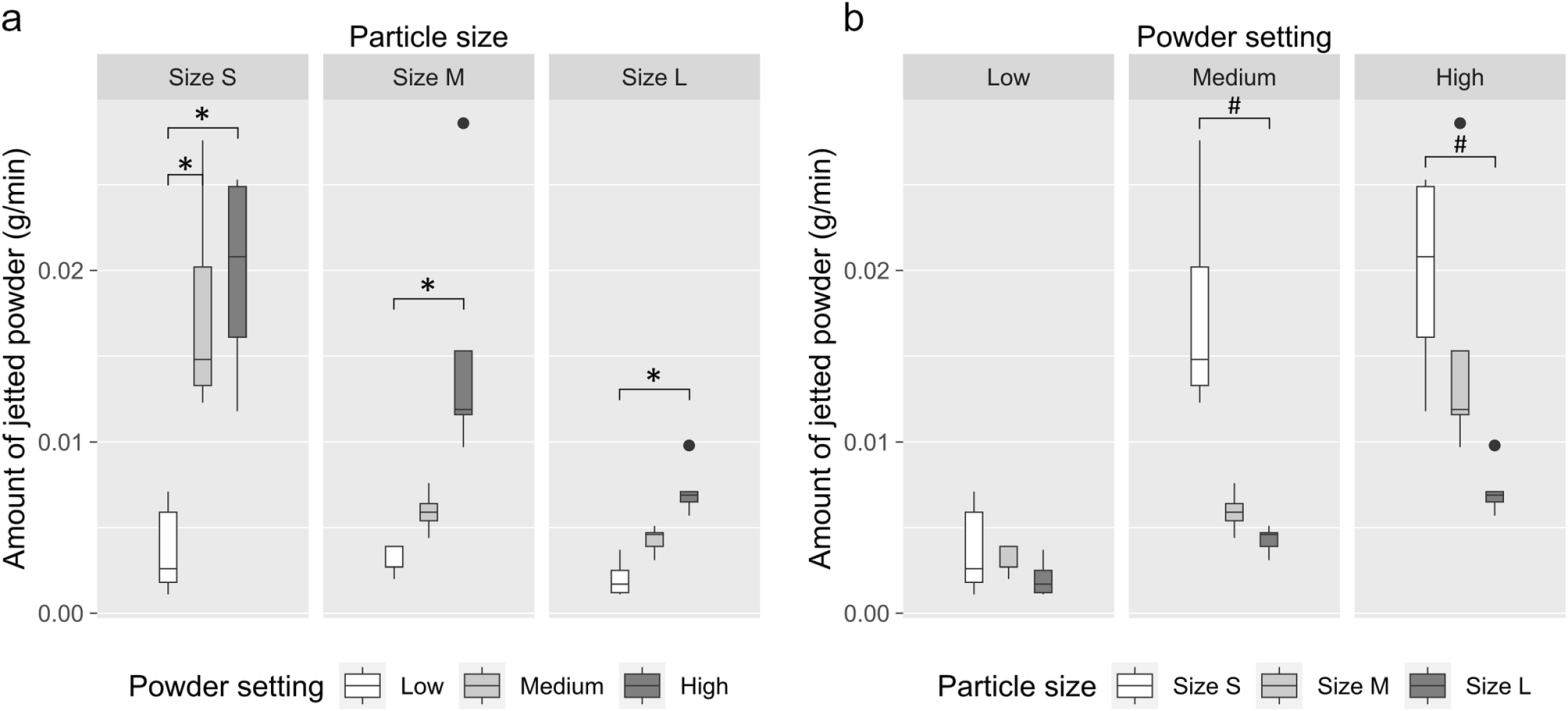
Measurements of the amount of jetted β-TCP powder (g/min). Compared between (**a**) different powder settings (low: white boxes, medium: light gray boxes, high: dark gray boxes) or (**b**) different sizes of β-TCP powder (size S: white boxes, size M: light gray boxes, size L: dark gray boxes). *p < 0.05 when compared between different settings; ^#^*p* < 0.05 when compared between different particle sizes.

Furthermore, the weight of jetted powder from different settings was determined and shown in Table 1. In general, there was a significant positive correlation between the amount of consumed powder (g/min) and strength of powder setting (consumed powder: high setting > medium setting > low setting) regardless of powder sizes by Kruskal–Wallis test. Dunn’s test showed that the differences were between high and low setting in sizes M and L. For size S the differences were between high and low, and medium and low strength settings (Fig. 4a). Regarding particle sizes, Kruskal–Wallis test showed statistically significant differences between sizes for medium and high strength setting (consumed powder: size S > size M > size L, all *p*-values < 0.05). Dunn’s test showed that these differences were between Size S and Size L for medium and high strength settings. For low strength setting no statistically significant differences between sizes could be seen (*p*-value > 0.05) (Fig. 4b).

**Table 1:** Average amount of consumed powder.

Amount of consumed powder (g/min)			
Powder setting	Size S	Size M	Size L
Low setting	0.0037	0.0030	0.0020
Medium setting	0.0176	0.0059	0.0043
High setting	0.0198	0.0154	0.0072

### Evaluation of cleaning effects by comparing different powder settings

**Figure 5:**
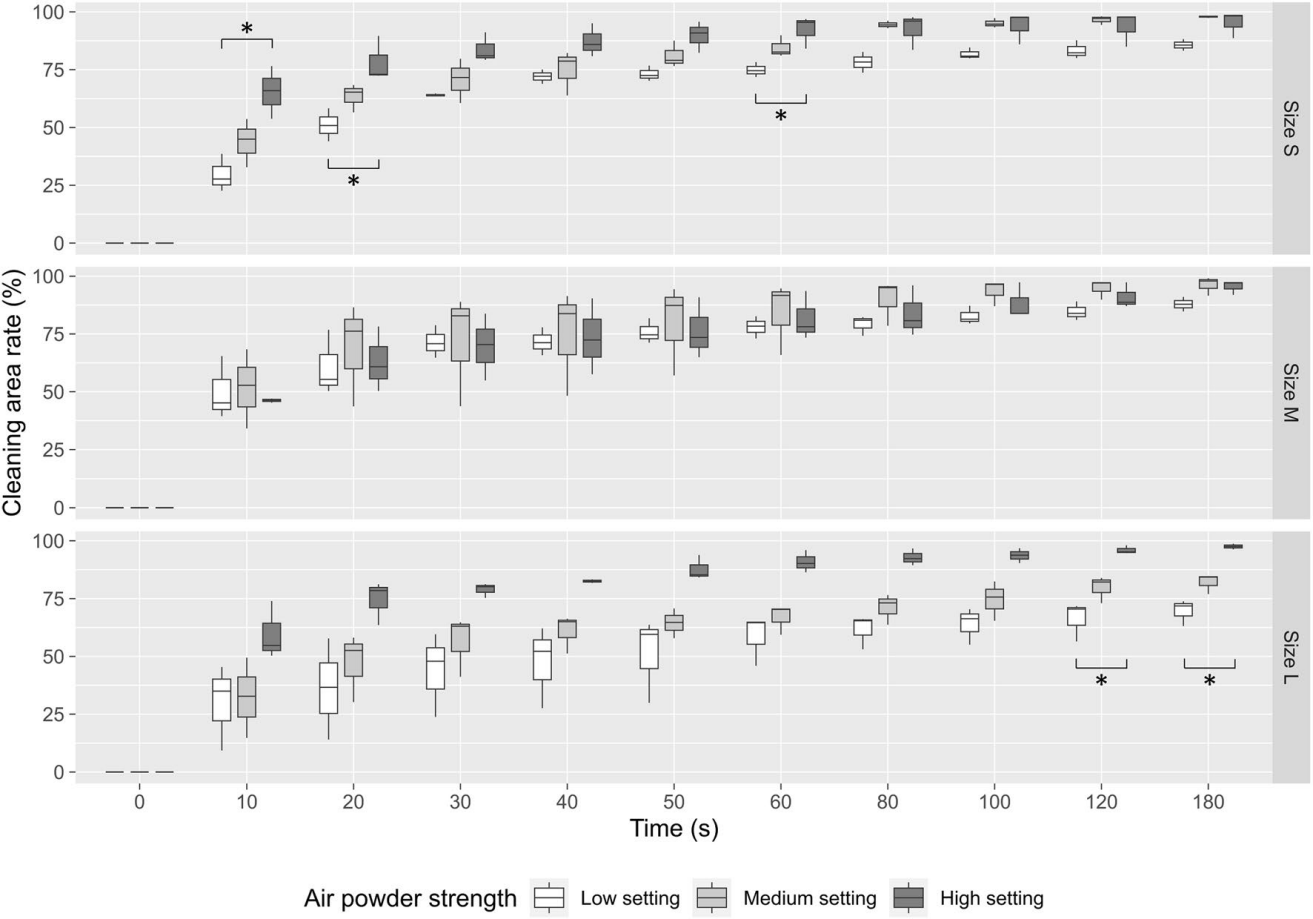
Comparison of cleaning capacity of APA treatment with different air powder strength (low: white boxes, medium: light gray boxes, high: dark gray boxes). Upper panel: size S; middle panel: size M; and lower panel: size L of β-TCP powder. *p < 0.05 when compared between different settings.

Next, the cleaning effects were evaluated by analyzing stain removal (simulating biofilm removal) from the implant surfaces after APA treatment. The first group of analyses examined the cleaning effects of different powder settings using the same size of β-TCP powder. For size S powder, the cleaning surface area percentage showed a significant difference depending on the powder setting at 10 s, 20 s and 60 s (Fig. 5, upper panel). The results of the cleaning rate showed that the cleaning ability was better when the powder setting was high during the cleaning procedure. Using the Kruskal–Wallis test, there was no significant difference between the three strength settings in the size M powder group (Fig. 5, middle panel). Interestingly, in the size L powder group, a difference between the low and high settings could be seen at the end of the cleaning at 120 s and 180 s (Fig. 5, lower panel).

### Evaluation of cleaning effects by comparing powder sizes

**Figure 6:**
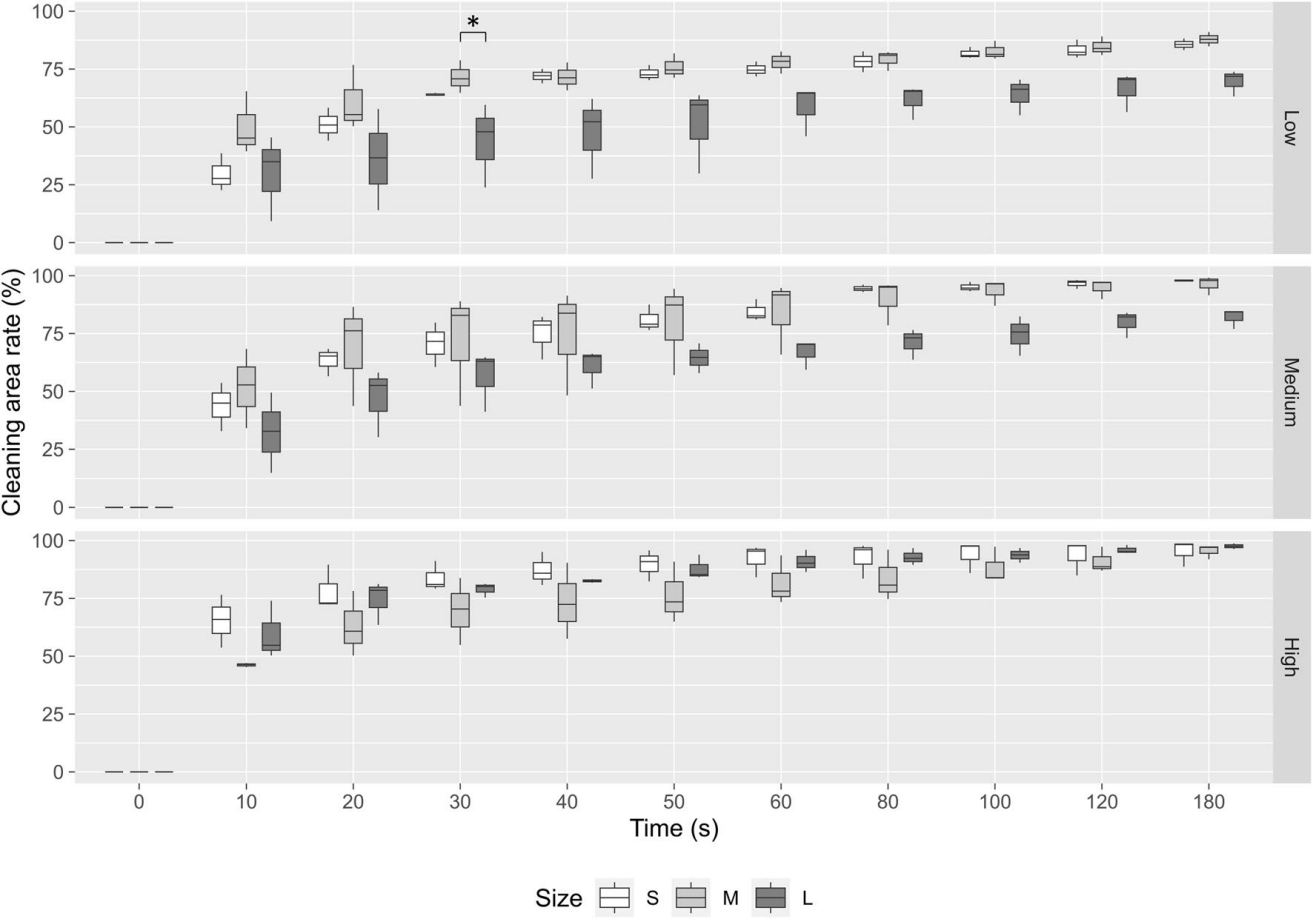
Comparison of cleaning capacity of APA treatment with different sizes of β-TCP powder (size S: white boxes, size M: light gray boxes, size L: dark gray boxes). Upper panel: low powder setting; middle panel: medium powder setting; lower panel: high powder setting. *p < 0.05 when compared between different particle sizes.

To investigate whether particle size would affect the cleaning ability, the cleaning rate was also analyzed by comparing different sizes of β-TCP powder under the fixed powder settings. From the data in Fig. 6, it can be seen that size S and size M powder (Fig. 6; size S: upper panel, size M: middle panel) were equally effective and presented better cleaning than size L powder at low and medium powder settings. However, at high powder setting, none of the differences measured were statistically significant between different sizes of β-TCP powder (Fig. 6, lower panel).

Furthermore, the time needed to clean 80% of the implant surface was presented in Table 2. Interestingly, data in this table can be compared with those in Table 1, which showed a clear trend of consumed powder and cleaning efficiency. It can be seen that at low setting with all size of particles and medium setting with size L, the jetted powder was less than 0.005 g/min and the time needed to clean 80% of the surface was longer than 60 s. By contrast, when the jetted powder was more than 0.005 g/min, the time needed to clean 80% of the surface was less than 60 s (size S and M for medium setting and all sizes for high setting, Tables 1 and 2), indicating the amount of consumed powder might be critical to the cleaning efficiency and 0.005 g/min of powder might be the threshold of cleaning effects in this study.

**Table 2:** Time needed to clean 80% of the implant surface.

Time to clean 80% of the surface (sec)			
Powder setting	Size S	Size M	Size L
Low setting	100 s	100 s	N.A
Medium setting	50 s	20 s	120 s
High setting	30 s	60 s	40 s

*N.A.* not available.

### Surface structure change of implants and sandblast and acid etching titanium discs

**Figure 7:**
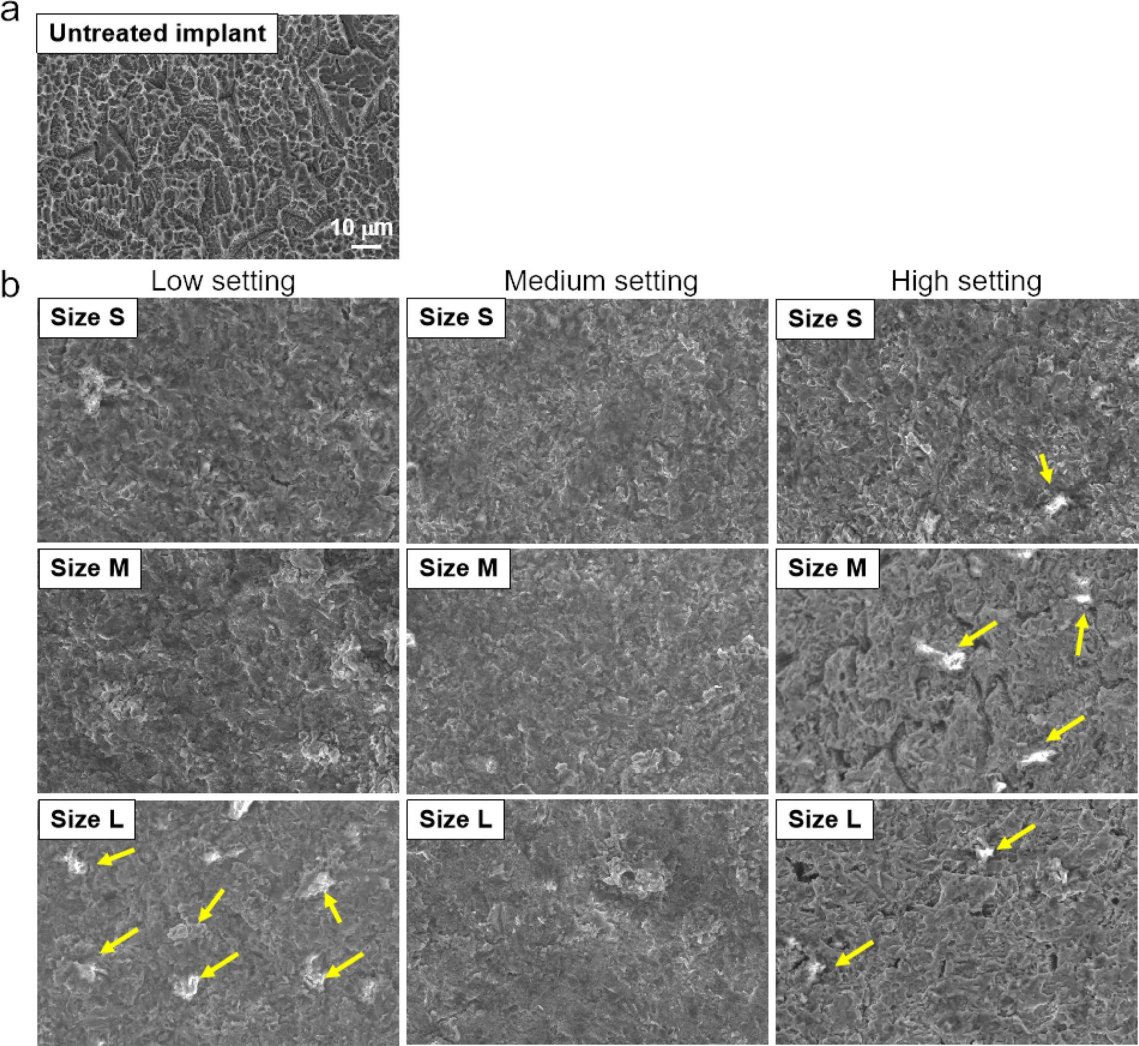
Images of the implant surfaces: (**a**) untreated control; (**b**) size S, M, L APA treatment for 180 s. Yellow arrows: particles left after cleaning.

APA treatment might result in changes on the implant surface^24^. To investigate the changes of the surfaces, APA treated implant surfaces were observed by SEM. Figure 7 presented the surface structure of implants treated by size S, M and L β-TCP with different strength settings. The results showed that APA treatment for 180 s altered the surfaces regardless particle sizes and jetting strengths. The white β-TCP particles in the images (yellow arrows) were left after cleaning and shown smaller in size than the original particles (Fig. 7b).

## Discussion

As mentioned in the review of literature, there are many studies that focused on the cleaning efficiency of APA treatment with powder by in vitro models. However, little to no data was found to connect the powder jetting strength, amount of jetted powder and particle sizes to the cleaning capacity, and only single powder jetting strength and fixed size of particle were tested in these investigations. The air pressure setting from different studies varied, ranging from 5.5 psi to around 80 psi (5.5 bar) and little information was understood if the powder jetting strength would affect the cleaning efficiency^22–24^. This current study is the first report to utilize various powder setting (low, medium and high) for jetting different sizes of particles and the strength of the settings was quantified by the load cell (from 14.71 to 18.75 g, corresponding to 6.75 to 8.6 psi roughly, Fig. 3). The cleaning capacity of different powder setting was systematically analyzed and we found that cleaning rate of high setting was in general higher regardless of particle sizes and it seemed likely that these results were in fact due to its larger jetting strength and the amount of jetted powder (Figs. 3, 4 and 5).

Moreover, this is also the first report providing a wide range of particle sizes for testing ink removal from APA treatment. Currently, glycine powder is a standard treatment option for peri-implantitis^15,37^. Prior to testing the cleaning effects of various β-TCP particles, we conducted a preliminary comparison of the cleaning rates between glycine and β-TCP particles (see Supplementary Fig. 1). The cleaning capacity of size S and M β-TCP particles was found to be similar to that of glycine after applying APA treatment for 180 s. From analyzing the cleaning rate according to particle sizes, the results showed that at low and medium powder settings, the cleaning capacity of size L particles (250 to 350 μm) was obviously lower than size M (105 to 117 μm) and S (53 to 105 μm) (Fig. 6, upper and middle panels). There is a possible explanation for these results that the amount of jetted size L powder was relatively less, resulting in a lower frequency to hit the surface. Only for high settings, the jetted powder reached 0.005 g/min regardless of particle sizes, attributing to the same cleaning capacity that size L particles could catch up with size M and S (Table 1, Fig. 6, lower panel). A study conducted by Matsubara et al. compared different sizes of powder including sodium bicarbonate (40 to 65 μm), glycine (25 μm) and erythritol (14 μm) for air abrasive treatment. The findings showed that sodium bicarbonate displayed greater cleaning capacity than glycine and erythritol because of its larger particle size^22^. However, the tested particle sizes were all relatively small and corresponded to size S (53 to 105 μm) in the current study. In addition, according to the other study by Tastepe et al., HA (5 to 35 μm) plus TCP (25 to 65 μm) or glycine powder (20 to 65 μm) can efficiently remove the biofilm from titanium surfaces^24^. Taken together, our results matched those mentioned in earlier studies and helped us understand how particle sizes could affect the cleaning capacity of APA treatment.

In addition to particle size and jetting strength, we also measured the amount of powder consumed during the APA treatment, which has not been reported in most related studies. Our analysis revealed an interesting finding: when the consumption rate exceeded 0.005 g/min, the cleaning time required to achieve 80% surface cleaning was less than 60 s (as shown in Tables 1 and 2). Previous research only reported the consumed powder amounts for HA and erythritol powders, which were 0.41 g and 0.54 g, respectively, to clean one titanium disc (10 mm in diameter) with 100% efficiency, taking an average time of 21 s^23^.

An ideal cleaning method should not change or damage the implant surface. However, the surfaces of implants and titanium discs changed in all treated groups and the size and type of particles seemed to influence the level of the change (Fig. 7 and Supplementary Fig. 2). To better understand the surface alternation after air polishing with β-TCP, sandblast and acid etching (SLA) surface titanium discs were used and abrasive treated with size S or size L β-TCP powder by high powder setting with different times. An untreated SLA disc displayed grooves and sharp edges (Supplementary Fig. 2a). The images showed that all APA treated surfaces changed according to the size of the particles. At 30 and 60 s, size S β-TCP abrasive treatment caused some visible changes and deeper grooves on the surface while rounded edges were observed by size L abrasive treatment (Supplementary Fig. 2b,2c, 30 and 60 s). However, the shaped edges around the grooves were rounded by size S or L β-TCP particles at 180 s, and no difference was observed between these two treatments (Supplementary Fig. 2b,c, 180 s). We propose that more of the smaller-sized particles may strike the surface per unit of time since the nozzle size was the same (Table 1). It has been reported that the larger diameter powder resulted in less damage to the surface. By measuring the depth and volume of defects produced in the teeth during the polishing process, glycine with a mean particle size of 100 μm caused less defects on the surfaces than the 63-μm-diameter glycine^38^. On the contrary, Matsubara et al. showed that larger-sized sodium bicarbonate (40 to 65 μm) caused more changes on titanium implant surfaces than glycine (25 μm) and erythritol (14 μm) by analyzing the surface roughness utilizing white light interferometry^22^. The other study compared the treated titanium disc surface after air polishing by TiO_2_, glycine, HA or HA plus TCP. The TiO_2_ group showed the least changes on the surface while HA and HA plus TCP groups presented more obvious flattened edges around the groove, and the possible explanation is HA particles are hard and round compared with TiO_2_ and TCP^24^.

An interesting result was observed in this study that APA treatment under medium powder setting (16.373 g) with size M β-TCP (105 to 117 μm) seemed to be the most efficient condition for cleaning. With this condition, it only took 20 s to clean 80% of the surface (Table 2) and only 0.0059 g/min of the powder was used (Table 1). One possible explanation is that at high powder settings, remnants of the powders could be detected as shown in Fig. 7, which may disturb the cleaning efficiency. Also, at the beginning of the treatment (30 s), size S β-TCP abrasive treatment already caused some visible deep grooves in the surface of the disc which might also disturb the cleaning efficiency (Supplementary Fig. 2b, 30 s). Additionally, the low powder setting of the machine could not drive enough particles to clean the surface for all sizes (Table 1, Fig. 4b). Nevertheless, these in vitro samples are different from the actual situation of the patients. It is indeed crucial to examine the cleaning method with biofilm to better simulate the actual clinical situation. Our ongoing investigation involves coating biofilm on implants or titanium discs using visible erythrosine dye. The initial and residual biofilm will be measured according to the procedure established in this study. Additionally, biofilm will be collected from specimens of volunteers with the approval of the ethics committee to simulate the real environment in the oral cavity. There is abundant space for further progress in verifying the setting of the parameters and the cleaning efficiency of this method by conducting animal or clinical studies to achieve cleaning purpose with minimal risks.

## Conclusions

This study set out to determine the factors that account for removing biofilm and cleaning implant surfaces by APA treatment. Based on these systematic comparisons, it is evident that the most effective method for cleaning implant surfaces might be by using size M particles (105 to 117 μm) with a medium setting. Implant surfaces were changed in all tested groups. We strongly believe that the aforementioned outcomes in this study might provide insight into the development of potential strategies to remove dental plaque and treat peri-implant diseases.

## Supplementary Information

Supplementary Figures.

## Data Availability

The data presented in this study are available on request from the corresponding author.
