# Supplementary material for: In vitro studies of factors affecting debridement of dental implants by tricalcium phosphate powder abrasive treatment
Source: Sci Rep. 2023 May 22;13:8271. doi: 10.1038/s41598-023-35053-3 (PMC10202908; doi:10.1038/s41598-023-35053-3)
Supplement: Supplementary file 1 — Supplementary Figures. [file 41598_2023_35053_MOESM1_ESM.pdf]

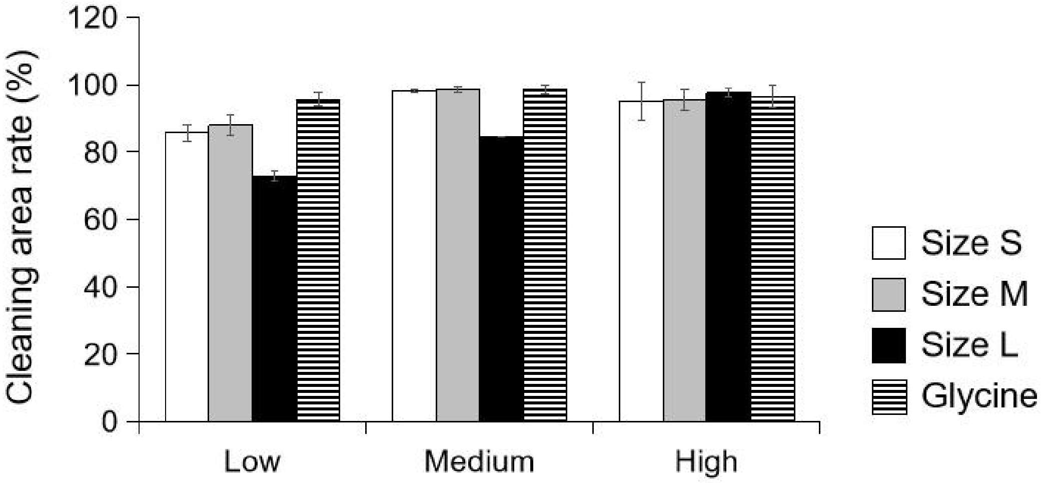

Supplementary fig 1. Cleaning capacity of APA treatment for 180 s with different air powder strength and different particles (white bars: size S of β-TCP; gray bars: size M of β-TCP; black bars: size L of β-TCP; horizontal stripes: glycine powder).

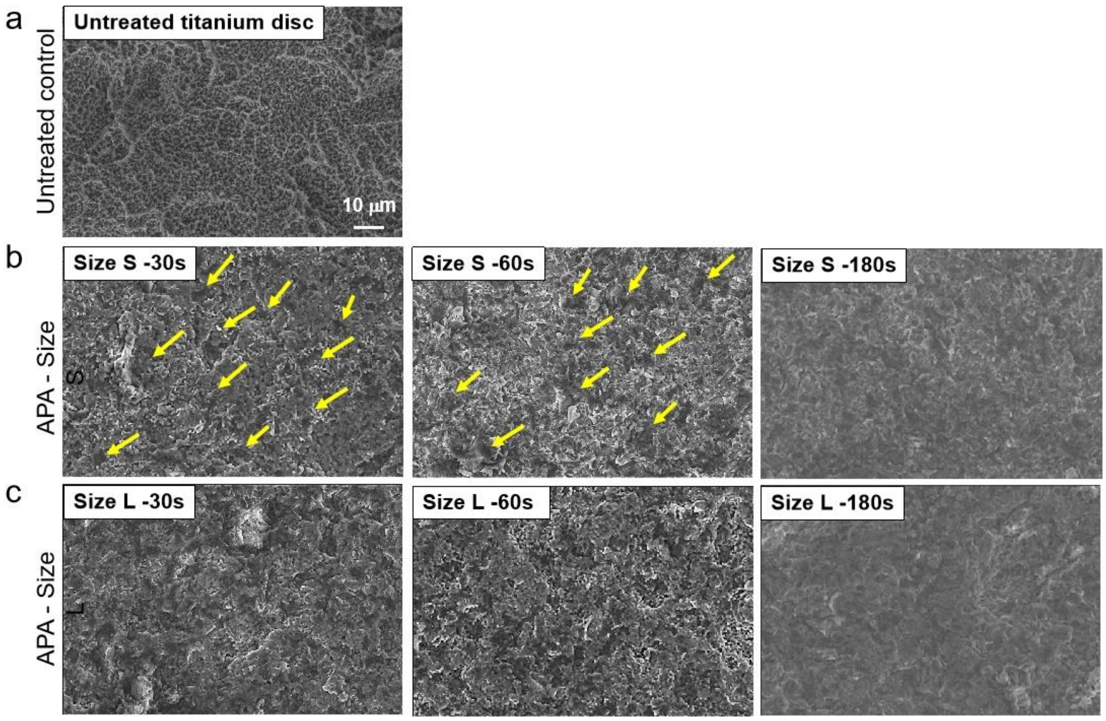

Supplementary fig 2. Images of SLA surface titanium discs: (a) untreated control; (b) size S APA treatment with high powder setting for 30, 60 and 180 seconds (yellow arrows: deep groove); (c) size L APA treatment with high powder setting for 30, 60 and 180 s.
